# Quantifying the Unitary Generation of Coherence from Thermal Quantum Systems

**DOI:** 10.3390/e21080810

**Published:** 2019-08-19

**Authors:** Shimshon Kallush, Aviv Aroch, Ronnie Kosloff

**Affiliations:** 1 Department of Physics and Optical Engineering, ORT-Braude College, 21982 Karmiel, Israel; 2 The Fritz Haber Research Center, The Hebrew University of Jerusalem, 91904 Jerusalem, Israel

**Keywords:** coherences, quantum control, quantum thermodynamics

## Abstract

Coherence is associated with transient quantum states; in contrast, equilibrium thermal quantum systems have no coherence. We investigate the quantum control task of generating maximum coherence from an initial thermal state employing an external field. A completely controllable Hamiltonian is assumed allowing the generation of all possible unitary transformations. Optimizing the unitary control to achieve maximum coherence leads to a micro-canonical energy distribution on the diagonal energy representation. We demonstrate such a control scenario starting from a given Hamiltonian applying an external field, reaching the control target. Such an optimization task is found to be trap-less. By constraining the amount of energy invested by the control, maximum coherence leads to a canonical energy population distribution. When the optimization procedure constrains the final energy too tightly, local suboptimal traps are found. The global optimum is obtained when a small Lagrange multiplier is employed to constrain the final energy. Finally, we explore the task of generating coherences restricted to be close to the diagonal of the density matrix in the energy representation.

## 1. Introduction

The existence of coherence in a system is a signature of its quantum properties. Coherence is considered as a resource in many applications [1,2]. Coherence underlies phenomena such as quantum interference and multipartite entanglement that play a central role in the applications of quantum thermodynamics and quantum information science [3,4]. In an energy representation a pure eigenstate $\phi_{j}$, $\hat{H}|\phi_{j}\rangle =\epsilon_{j}|\phi_{j}\rangle$ has no coherence. The maximally coherent pure state is $|\Psi \rangle =\frac{1}{\sqrt{d}}\sum_{j-0}^{d-1}|\phi_{j}\rangle$, where *d* is the dimension of Hilbert space [2].

The notion of coherence can be generalized to open quantum systems with non pure states. A stationary state which commutes with the Hamiltonian $[\hat{H},{\hat{\rho }}_{st}]=0$ has no coherence. A primary example is a thermal state $\rho_{T}=\frac{1}{Z}e^{-\beta \hat{H}}$ diagonal in the energy representation, where $\hat{H}$ is the Hamiltonian of the system, $\beta =\frac{1}{k_{B}T}$ and *Z* is the system’s partition function: $Z=\mathrm{Tr}\{e^{-\beta \hat{H}}\}$. In addition, a thermal state is passive, characterized by a monotonic decreasing probability distribution of the occupation of its energy levels [5,6,7].

States with coherence are dynamically evolving due to the non-diagonal elements of their density matrix. A signature of the coherence can be observed by variables that do not commute with the Hamiltonian. Examples of such observables are molecular alignment and orientation [8,9,10].

The issue addressed in this study is the optimal generation of coherence from an initial thermal state. In the context of molecular alignment, for example, this is related to the question what is the upper bound for the utmost alignment that could be created by external coherent field, starting from a thermal ensemble with a given temperature. The specific question of creating the utmost alignment and orientation, or even more generally, the upper and lower bounds for a given control task have been investigated [11,12].

The generation of coherence is intimately connected to the operation of quantum heat engines [13]. In reciprocating quantum engines, finite power requires fast dynamics on the adiabatic strokes generating coherence. The accompanying decoherence on the thermal strokes has been termed quantum friction [14,15]. In the limit of short cycle times or for continuously operating engines coherence is essential [16].

Generating coherence from a passive state involves a cost in work. This is because in unitary evolution energy excitations are required to allow for coherence. A limiting case is adiabatic evolution which will maintain a passive energy state with no coherence. The trade-off between invested work and coherence generated has been noticed [15,17,18]. In this study we search for a unitary control scheme leading to a state with the optimal attainable coherence. In addition, we quantify the cost in work for the optimal solution [19].

Coherence is associated with off-diagonal elements of the density operator *in the energy representation*. There have been several suggestions to quantify the amount of generated coherence [20,21]. In this study we will use a distance metric D and C (see below). With these definitions we can first define a unitary control task to achieve maximum coherence. Then we can check if the control achieves the upper limit of coherence. In addition obtain the work cost associated with this task.

The present study incorporates the theory of quantum control to address a quantum task of optimizing coherence. The fulfilment of this task depends on the constraints imposed: Unconstrained optimization, (Section 3) maximum coherence with minimum energy invested (Section 4) or limiting the coherence to be close to the diagonal (Section 5).

## 2. General Aspects of Coherence Measurment: Information and Entropy

We study the creation of coherence from a passive state by a unitary evolution generated by the control Hamiltonian $\hat{H}$:

(1) $$\hat{H}\left(t\right)={\hat{H}}_{0}+\hat{\mu }\cdot f\left(t\right)$$

where ${\hat{H}}_{0}$ is the stationary Hamiltonian, $\hat{\mu }$ is the control operator, f(t) is the time dependent control field. For most purposes the field vanishes at the initial and final time so that: f(0)=f(T)=0. The dynamics generated by the control Hamiltonian are given by the unitary evolution operator:

(2) $$i\hbar \frac{d}{dt}{\hat{U}}_{c}=\hat{H}\left(t\right){\hat{U}}_{c}\;\;\;\;\;\;{\hat{U}}_{c}\left(0\right)=\hat{I}.$$

We assume complete controllability meaning that the control field can generate any unitary transformation ${\hat{U}}_{c}$ in the Hilbert space of the system. We seek a unitary ${\hat{U}}_{c}$ which will transform an initial thermal state ${\hat{\rho }}_{T}$ to a final state ${\hat{\rho }}_{f}$ with maximum coherence

(3) $${\hat{\rho }}_{f}={\hat{U}}_{c}^{†}{\hat{\rho }}_{T}{\hat{U}}_{c}$$

We now need to quantify the coherence.

The Shannon entropy [22] associated with a complete measurement of the observable $\langle \hat{A}\rangle$ is

(4) $$S_{A}=-\sum_{j}p_{j}\ln p_{j}$$

where $p_{j}=\langle {\hat{P}}_{j}\rangle =\mathrm{Tr}\left\{\hat{\rho }{\hat{P}}_{j}\right\}$ is the probability of outcome *j* considering the spectral decomposition of the operator $\hat{A}=\sum \alpha_{j}{\hat{P}}_{j}$. A special case is the energy entropy $S_{E}$ associated with a complete measurement of $\hat{H}$. In information theory terms, the entropy with respect to a variable quantifies the amount of classical information obtained by a complete measurement *with respect to the variable*. If the evolution operator $\hat{U}$ does not commute with $\hat{A}$ the entropy of the observable $S_{A}$ will change.

An invariant to the evolution is the von Neumann entropy $S_{vN}$ [23]:

(5) $$S_{vN}=-\mathrm{Tr}\left(\hat{\rho }\ln\hat{\rho }\right)$$

Due to its invariance with respect to unitary transformations $S_{vN}$ is often used to quantify the *total* information content of quantum systems [24]. As shown in Reference [25], $S_{vN}$ is the minimal entropy that could be obtained by a complete measurement of all possible operators. Therefore, $S_{vN}\leq S_{A}$. For a thermal state ${\hat{\rho }}_{T}$ the energy entropy and the von Neumann entropies coalesce $S_{E}=S_{vN}$.

A quantifier of distance between two states ${\hat{\rho }}_{a}$ and ${\hat{\rho }}_{b}$ is the divergence [26]:

(6) $$D\left({\hat{\rho }}_{a}|{\hat{\rho }}_{b}\right)=\mathrm{Tr}\left\{{\hat{\rho }}_{a}\ln{\hat{\rho }}_{a}-{\hat{\rho }}_{a}\ln{\hat{\rho }}_{b}\right\}$$

$D({\hat{\rho }}_{a}|{\hat{\rho }}_{b})\geq 0$ with equality when ${\hat{\rho }}_{a}={\hat{\rho }}_{b}$. A measure of coherence is the distance between a state $\hat{\rho }$ and ${\hat{\rho }}_{E}$ where ${\hat{\rho }}_{E}$ is the diagonal part of the density operator $\hat{\rho }$ in the energy representation. We can quantify this distance by $D_{E}\left(\hat{\rho }|{\hat{\rho }}_{E}\right)$ so it becomes the difference between the von Neumann entropy and the energy entropy [27,28]:

(7) $$D\left(\hat{\rho }|{\hat{\rho }}_{E}\right)=S_{E}-S_{vN}$$

For a thermal state ${\hat{\rho }}_{T}$ the divergence vanishes. Under unitary transformation $S_{vN}$ is conserved but the energy entropy $S_{E}$ can increase and with it the divergence $D(\hat{\rho }|{\hat{\rho }}_{E})$, therefore maximizing $S_{E}$ will lead to maximum coherences.

An alternative direct measure for coherences is:

(8) $$C=\frac{2N}{N-2}\sum_{i,j>i}{\left|\rho_{ij}\right|}^{2}$$

where the sum of the magnitude of the off diagonal elements of $\hat{\rho }$ in the energy representation is measured. C is normalized such that C=1 is obtained for a fully coherent pure state.

## 3. Maximum Coherences I: The General Case and Its Relation to the Micro-canonical Ensemble

We start by analyzing the maximum generated coherences in an unconstrained case. The entropy is a monotonic function of the temperature. It is zero for T=0 and the thermal state with the maximum (either von Neuman and Shannon) entropy is the microcanonical ensemble in which $p_{j}=1/N$ and $S_{vN}=S_{E}=\ln N$. This corresponds to a state with effective T→∞. Now, any initial thermal state with lower *T* will have lower $S_{E}$. Therefore the unitary transformation that will maximize $D(\hat{\rho }|{\hat{\rho }}_{E})$ is the one that transforms ${\hat{\rho }}_{T}$ into a *population distribution* similar to the microcanonical ensemble on the energy diagonal but, unlike the micro-canonical thermal state, with non-diagonal coherences. The absolute maximal generated coherences for any system is therefore starting from an initial pure state, which for a thermal state with T=0 is the ground state and vanishing entropy $S_{vN}=0$.

### Computational Control Demonstration and Model

For the sake of generic and scalable demonstration we employ here the model of a many body single mode Bose-Hubbard double well system [29]. This many-body Hamiltonian for *n* atoms is equivalent to a system of N=2j+1 sub-levels with angular momentum j=n/2 [30]. The Hamiltonian model becomes:

(9) $${\hat{H}}_{0}=U{\hat{J}}_{z}^{2}+\Delta {\hat{J}}_{x}$$

where ${\hat{J}}_{i}$ are the projections of the total angular momentum on the *i* axis. *U* is the in-site inter-particle interactions parameter and Δ is the hoping strength between the two wells. Both are set hereby to one. Adding the control operator ${\hat{J}}_{z}$ makes this Hamiltonian completely controllable—for more details, see Reference [30]. To enable systematic comparison between systems with different sizes, the energy of the system is normalized so the effective free Hamiltonian of the system is taken as ${\hat{H}}_{n}={\hat{H}}_{0}/\mathrm{Tr}\left({{\hat{H}}_{0}}^{2}\right)$.

**Figure 1:**
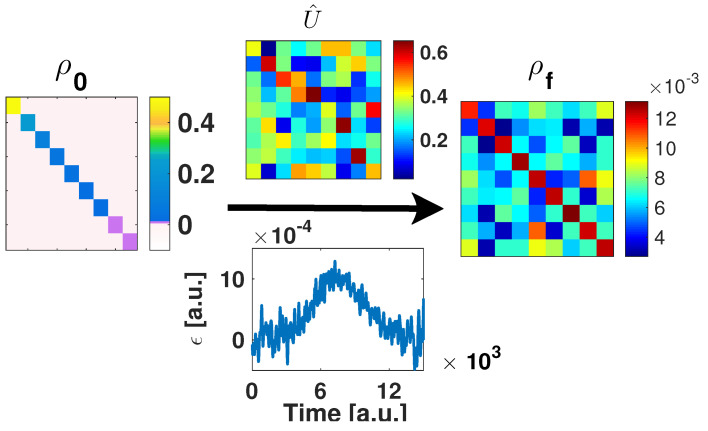
Unitary transformation generating maximum coherence: Complete controllability. Results of the Optimal-Control-Theory field that was generated to take the thermal state with j=4 (N=9) and $\beta_{0}=0.2$ with the Hamiltonian of Equation (9) into the micro-canonical population distribution. (**Left**) panel: initial thermal density matrix. (**Upper**) and (**lower**) panels: absolute values of the elements of the obtained unitary transformation and its generating field, respectively. (**Right**) panel: absolute values for the transformed density matrix.

Complete controllability [31] means that one can found a field that generates the desired unitary transformation. To illustrate this ability on the current context, we use the Hamiltonian of Equation (9), with the initial thermal state with inverse temperature $\beta_{0}=1/k_{b}T_{0}=0.2$1/Hartree. The control hamiltonian $\hat{H}={\hat{H}}_{n}+\epsilon \left(t\right){\hat{J}}_{z}$ was then used to generate the target unitary transformation. This unitary operator transforms the thermal state into the micro-canonical distribution on the diagonal. By construction it leads to maximum coherence. Figure 1 presents an image matrix-plot for the initial and the final absolute values of the density matrices, the computed unitary transformation and the field ϵ(t) that produced it.

The optimality of the micro-canonical probability distribution with respect to coherences, is demonstrated by the following numerical optimization: Starting with given ${\hat{\rho }}_{T}$ with corresponding $\beta_{0}=1/k_{b}T_{0}$, find the unitary transformation: ${\hat{U}}^{op}=\exp\left(i{\hat{V}}^{op}\right)$ such that the functional $D(\hat{\rho }|{\hat{\rho }}_{E})$ is maximum. For an *N* level system, this defines a control problem with dimensionality N×N free control parameters, defined by the hermitian matrix ${\hat{V}}^{op}$. Note that the object for this control problem is the unitary transformation itself. Since the system is controllable we implicitly assume the existence of a field that generates it. An explicit example is demonstrated in Figure 1.

**Figure 2:**
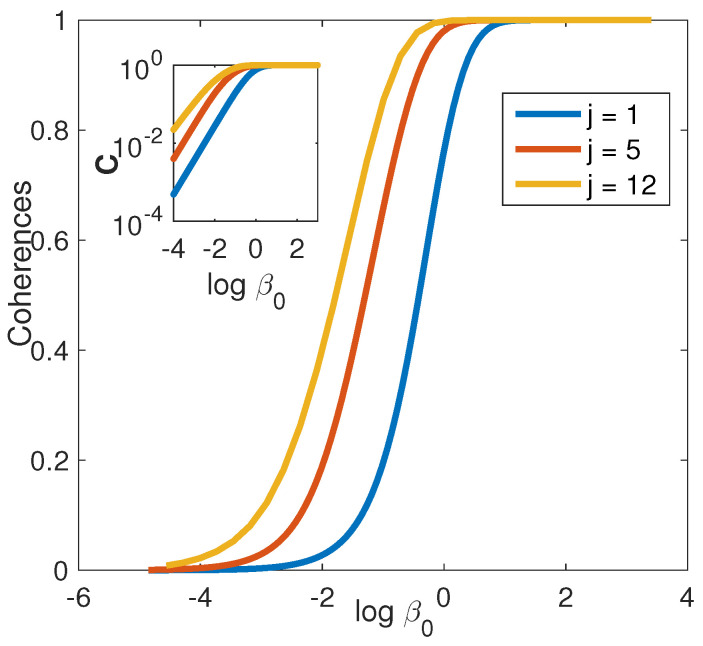
Micro-canonical ensemble: Maximal coherences defined by Equation (8), as a function of the initial verse temperature β, for different sizes of the system. Inset: A log-log plot of the main panel.

Figure 2 presents the value of the direct optimal total coherences measure C defined in Equation (8), as a function of the logarithm of the inverse temperature $\beta_{0}$ for different sizes of the system. For all cases, the optimal population distribution was found to be the microcanonical ensemble. As expected, for increasing temperature, the level of coherence generated decrease smoothly so that at T→∞ no coherence is generated, vanishing exponentially with the initial $\beta_{0}$ (see left inset). For larger systems, coherence is generated for higher initial temperatures. Note that the optimum is highly degenerate, represented by a N(N−1)/2-fold dimensional sphere with a vanishing radius for T→0 and T→∞ and a maximum in an intermediate β. Being loosely constrained and highly degenerate, the optimization is found to be globally trap-less. That is, for all the sizes of the system and for any initial guesses for the unitary transformation, no suboptimal solutions were found. All the solutions converged to the global optimum of the micro-canonical distribution $p_{i}=1/N$. Difficulties to converge to the optimum were found for j>12 (N=25). The extremely large dimensionality of the optimization problem (above 625 optimization parameters) cause difficulty in computing the numerical gradient. We have to remark here, that despite of being related to the general context of traps in the control space of parameters, this finding is entirely different from the notion of the no trap theorem [32,33]. The theorem deals with the landscape for the problem of finding a giving field for some desired transformation. This is the case for most of the Optimal Control Theory related works in quantum coherent control. In this work, however, the optimization target is the transformation itself.

## 4. Maximum Coherence II: Energy Constraint and The Canonical Probability Distribution

The micro-canonical probability distribution on the diagonal is the state with utmost coherences for a *N* level system. This state, however, corresponds to a uniform population distribution, which effectively matches infinite temperatures for the probability distribution. The natural constraint in addition is a target with finite energy.

The state with maximum entropy constrained by an average energy $\langle \hat{H}\rangle =E$ is the thermal state ${\hat{\rho }}_{T}$ and its entropy $S_{vN}=S_{E}=\ln Z+\beta E$ [34]. For T→∞ the thermal state converges to the micro-canonical state ${\hat{\rho }}_{T}\to {\hat{\rho }}_{mc}$. For a state with finite *T* the maximum entropy is obtained for *population* which corresponds to the Boltzmann distribution. Therefore, a unitary transformation with a target state where the populations are canonically distributed will maximize coherences for a predetermined final energy. Consequentially, any initial thermal incoherent state, is minimal with respect to its entropy. Generating coherences requires an investment of energy. The process is reversible, nevertheless returning to the original energy will necessarily erase the coherences. Moreover, for any passive states, this also means that the energy of a thermal state cannot be reduced by a unitary transformations due to the fact that coherences cannot be produced.

### Numerical Example

The constraint on the final energy is introduced by a Lagrange multiplier in addition to the previous optimization of the divergence. The optimization problem is now in the following form: Starting with with the inverse temperature $\beta_{0}$ and ${\hat{\rho }}_{T}$, find the optimal unitary transformation: ${\hat{U}}^{op}=\exp\left(i{\hat{V}}^{op}\right)$ such that $J=S_{E}-\lambda \left|E-E_{f}\right|$ is maximal. λ is the Lagrange multiplier that imposes the energy constraint.

**Figure 3:**
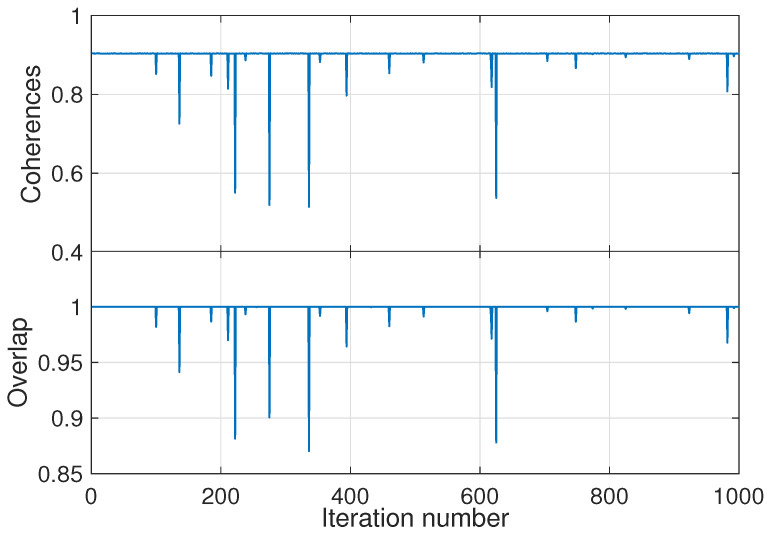
Canonical ensemble. (**Upper**) panel: The values for C for unsorted 1000 optimization runs. The parameters for the runs were taken as j=1, the initial temperature is β=3 and the energy of the final state corresponds to $E_{f}=1/k\beta_{f}$ were $\beta_{f}=0.3$. (**Lower**) panel: Overlap between the final populations of the density matrix and the target thermal state. $\lambda =0.3=\lambda_{0}$ (see text) is taken.

The optimization with the additional constraint leads to traps: many local suboptimal solutions. We use this feature to corroborate the result of this section. Figure 3 presents in the upper panel the measure of the coherence C for 1000 optimization runs with different initial random guesses for the unitary transformation. At the bottom panel the overlap between the diagonal part of the final density matrix $p_{i}^{f}$ and the thermal population at the target energy $p_{i}^{T}$, defined by: $O=\sum_{i}\sqrt{p_{i}^{T}p_{i}^{f}}$, is shown. This measurement is, in a way, a reduction of the conventional overlap between two wavefunctions to the incoherent case.

The suboptimal traps that were obtained for runs with values of coherences below the maximum ones are clearly visible. Moreover, the correspondence between the traps in the plots is strict and, as expected, a complete overlap with the target thermal distribution also leads to a maximum in the coherences.

**Figure 4:**
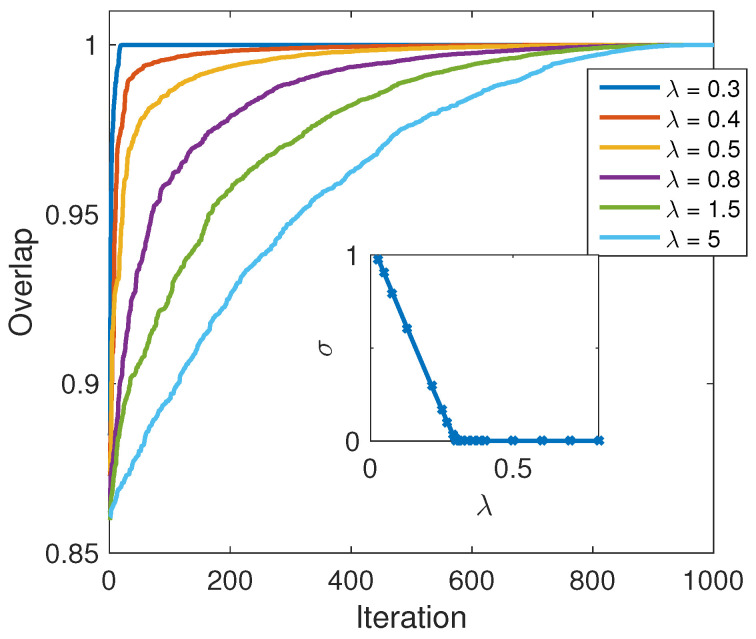
λ and number of traps. Main panel: Sorted overlap (see main panel of Figure 3) for 1000 runs, for different values of the Lagrange multiplier values λ. Inset: the mean error in the resulted energy as a function of the Lagrange multiplier λ.

Remarking on the number of traps in the system: The inset in Figure 4 shows the averaged relative error in the final energy of the state σ as a function of the Lagrange multiplier λ:

(10) $$\sigma =\frac{E-E_{T}}{E_{T}}$$

where *E* is the actual energy of the final state and $E_{T}$ is the target thermal energy. For very small λ→0, the energy constraint is too weak to impact the optimization and the solution does not converge to the correct target energy. However, violation of the energy constraint decreases linearly with λ, probably due to the linearity of the energy in the constraint. Adequate numerical tolerance was reached for some $\lambda_{0}$. The main panel of the figure show the sorted values for the overlap between the final density matrix and the thermal target, for 1000 optimization runs and different values of λ. For the case presented here $\lambda_{0}=0.3$, it is interesting to see that the number of traps is minimal very close to $\lambda_{0}$ and increases monotonically with the increase of the Lagrange multiplier. This is a result of the relative weight of the optimized quantity D which is relatively decreased, resulting in suboptimal overlap and coherences.

## 5. Additional Constraints Imposing Coherence Predominately Close to the Diagonal

Typically in controllable problems the control operator is biased to couple adjacent energy levels. For example, the electric dipole or the polarizability tensor operators [35]. In the context of molecular spatial directions for example, both operators are related to cosθ and cos${}^{2}$θ, where θ is the angle between the molecular and spatial axis. The two kinds of interactions lead to molecular orientation and alignment, respectively. These light-matter coupling operators connect directly only adjacent (or next-to-adjacent) *j* levels. Hence, coherences will be first generated at near *j* proximity. Higher order of coupling, between distant energy levels, are not forbidden but they are harder to achieve and are not measured under conventional alignment or orientation experiments. In control terminology, the problem of creating highly non local coherences by local operators is still controllable but is not invertible, that is, the solution for the control problem will be very hard or even unfeasible.

**Figure 5:**
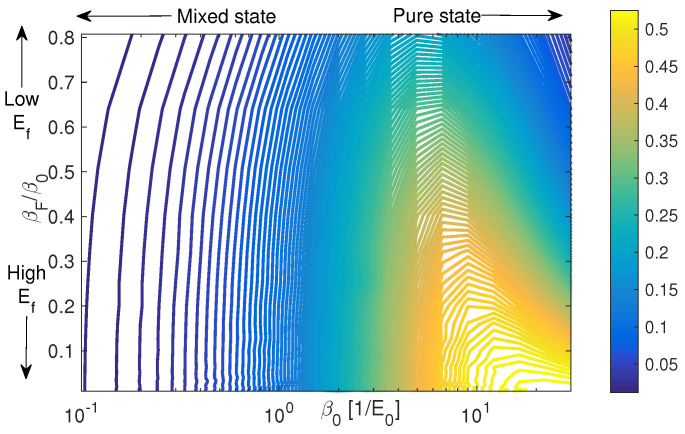
Coherences and phase space proximity I. Maximal values for the generalized coherences operator $\hat{O}$, defined in Equation (11), as a function of the initial temperature $\beta_{0}$ and final effective temperature $\beta_{F}$. The other parameters taken here are α=0.04 and j=3.

In this section the relation between differently remote coherences and their structure will be studied. The optimization is modified, with the target being now:

(11) $$\hat{O}=\exp\left(\alpha \hat{\mu }\right)-diag\left(\exp\left(\alpha \hat{\mu }\right)\right)$$

where

(12) $$\hat{\mu }=\left(\begin{array}{ccccc} 0 & 1 & & & \\ 1 & 0 & 1 & & \\ & 1 & \ldots & \ldots & \\ & & \ldots & & 1\\ & & & 1 & 0 \end{array}\right)$$

is a simplified dipole operator. Under these definitions, $\langle \hat{O}\rangle$ is a measure of the amplitude of the off-diagonal coherences. For small values of α, $\hat{O}\to \hat{\mu }$ only adjacent coherences are taken into account and for increasing α the whole matrix is covered, merging into the micro canonical ensemble result of the previous section. The control problem now is to find the unitary transformation that find the maximal expectation value for the operator $\hat{O}$, for some initial temperature and a given final energy constraint. Figure 5 displays a contour plot of the optimal expectation value of $\hat{O}$ under the unitary transformation that gives optimal $\langle \hat{O}\rangle$ for the system with j=3 (N=7) and α=0.04. The values are plotted as a function of the initial temperature $\beta_{0}$ and the final energy defined by the effective temperature $\beta_{F}$. The *x*-axis values are an indication of the initial purity of the system, so that the system is pure for $\beta_{0}\to \infty$ and highly mixed for vanishing $\beta_{0}$. The *y*-axis is an indication for the resulted energy of the system. The optimization problem in this case contains, of course, many traps, and each of the points in the contour plot is globally optimal, verified by initiating the optimization with 10,000 random guesses for the transformation and choosing the (converged) maximum value that was obtained for $\hat{O}$. The maximum of the coherence generated is obtained for an initial pure state that is transformed to final infinite temperature and distribution on the diagonal, that is, the micro-canonical ensemble.

**Figure 6:**
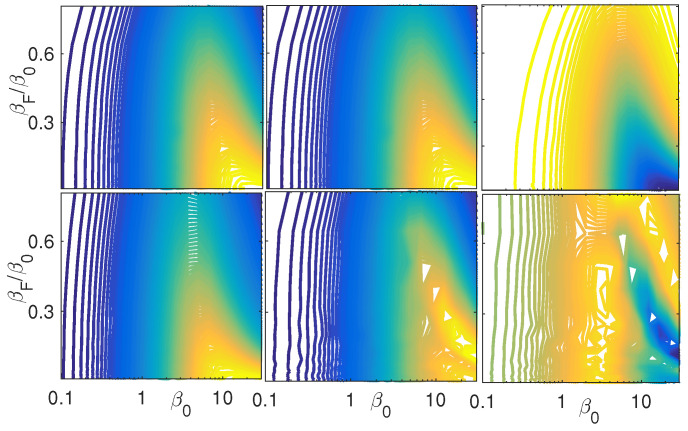
Coherences and phase space proximity II. Upper panels: Similar to Figure 5, plotted in the upper left panel reference. The middle and right upper panels are the expectation values of $\hat{\mu }$ and ${\hat{\mu }}^{2}$ at the optimal conditions that were obtained for the left upper panel of the operator $\hat{O}$. The rest of the parameters are similar to Figure 5. Lower panels: Same as the upper panels, with α=40.

Figure 6 expands the result of Figure 5 to other regimes and operators. As a reference, the results of Figure 5 are displayed on the upper left panel of the figure. The middle upper panel displays the expectation values of $\hat{\mu }$ of the target state which optimizes $\hat{O}$. For this case α=0.04≪1 the optimization of the two operators converges to the same outcome. The right upper panel of figure shows the same dependency, under the same conditions, for the non-diagonal part of the operator ${\hat{\mu }}^{2}$, which quantifies the second row off-diagonal elements. It is interesting to note that the maximum conditions for coherences for $\hat{\mu }$ is associated with minimal coherences for ${\hat{\mu }}^{2}$. This feature of inverted extremum seems to hold also for higher orders of $\hat{\mu }$. The lower panels of Figure 6 present the same veriables of the upper panel but here α=40≫1. For this case, the maximum of $\hat{O}$ is still found for an initial pure state and maximum final energy, but the entire structure does not overlap with the conditions for the linear dipole coherences. The structure that flips the signs of the minimum and maximum with the order of $\hat{\mu }$ is nevertheless maintained. As a final remark, it is worth mentioning that the same procedure was tested also for a generator of the form ${\hat{O}}_{2}\propto \exp\left(\alpha \mu^{2}\right)$. For this generator the optimal transformations for ${\hat{O}}_{2}$ were found to contain only coherences of even order in $\hat{\mu }$ and the inversion of the minimum and maximum takes place now with ${\hat{\mu }}^{2}$.

## 6. Summary

In this work the ability to induce coherence into systems by means of an external control field was investigated. We found that for any thermal state the coherence generated will become maximal when the system is transformed into a population distribution that matches the microcanonical energy distribution. Adding an energy constraint to the final target state leads to an optimal population distribution that matches the canonical distribution. Note that the maximum coherence could be also obtained indirectly by imposing a complete *population transformation* target [36] on the optimization procedure instead of the full unitary transformation.

Finally, it is worth while to mention that the insight obtained from the control problem is simple and robust, nevertheless their physical realization is non trivial. Even where the system is controllable, the actual typical operators that generate the transformation have low connectivity within the whole Hilbert space. In that case it is difficult to generate coherence between distant energy states. The last section was devoted to this restriction, and the interesting properties and symmetries that emerge between differently remote states were discussed. The present study is a link between quantum control and quantum thermodynamics [3,37] pointing to the work cost required to generate coherence.
